# Supplementary material for: Genetic Potential and Inheritance Patterns of Physiological, Agronomic and Quality Traits in Bread Wheat under Normal and Water Deficit Conditions
Source: Plants (Basel). 2022 Mar 31;11(7):952. doi: 10.3390/plants11070952 (PMC9002629; doi:10.3390/plants11070952)
Supplement: Supplementary file 1 [file plants-11-00952-s001.zip › plants-1605022-supplementary.pdf]

Table S1. Code, name, pedigree and source of the eight parental wheat genotypes used in the present study.

Name	Code	Pedigree	Source
Gemmeiza-12	P_1	OTUS /3/ SARA / THB // VEE	ARC-Egypt
Sids-12	P_2	BUC // 7C / ALD /5/ MAYA74 / ON // 1160.147 /3/ BB / GLL /4/ CHAT"S" /6/ MAYA / VUL // CMH74A.630 / 4*SX	ARC-Egypt
Misr-2	P_3	SKAUZ / BAV92	ARC-Egypt
Line-144	P_4	BAJ #1	CIMMYT-Mexico
Line-128	P_5	SOKOLL/3/PASTOR//HXL7573/2*BAU/4/SRMA/TUI	CIMMYT-Mexico
Sahel-1	P_6	NS 732/PIMA//Veery'S	ICARDA
Gemmeiza-7	P_7	CMH 74A.630 / 5X // SERI 82 /3/ AGENT	ARC-Egypt
Line-121	P_8	CHEN/AE.SQ//2*OPATA/3/FINSI	CIMMYT-Mexico

Table S2. Main soil physico-chemical analysis before wheat cultivation at the experimental site.

Depth (cm)	Sand (%)	Silt (%)	Clay (%)	texture	θ_s (%)	LL (%)	DUL (%)	OC (%)	ϱb (g cm^{-3})	Ks (cm h^{-1})
0-20	16.0	30.1	53.9	clay	62.7	22.0	40.6	0.8	1.20	0.40
20-40	15.5	32.5	52.0	Clay	65.2	21.5	40.5	0.7	1.22	0.38
40-60	14.5	37.0	48.5	clay	65.5	21.0	41.2	0.5	1.23	0.31

	pH	Available N (mg kg^{-1})	Available P (mg kg^{-1})	Available K (mg kg^{-1})	OM (%)
0-20	8.0	35.2	10.5	620	1.37
20-40	7.8	32.5	9.5	602	1.21
40-60	8.2	28.9	9.2	585	0.86

θ_s: saturation percentage (%), LL: wilting point (%), DUL: field capacity (%), OC: organic carbon (%), ϱb: bulk density, K_s: saturated hydraulic conductivity, OM: organic matter

Table S3. Drought tolerance indices for wheat parental genotypes and their corresponding F1s crosses.

Gen	Geometric mean productivity (GMP)	Mean productivity (MP)	Yield index (YI)	Stress tolerance index (STI)
P1	24.92	25.43	0.91	0.70
P2	24.58	24.83	0.96	0.68
P3	28.87	29.13	1.13	0.94
P4	26.60	26.90	1.03	0.80
P5	20.67	21.03	0.77	0.48
P6	25.28	25.42	1.02	0.72
P7	20.98	21.33	0.78	0.49
P8	28.46	28.63	1.14	0.91
P1×P2	25.91	26.54	0.93	0.75
P1×P3	26.42	26.69	1.03	0.79
P1×P4	27.37	27.72	1.05	0.84
P1×P5	27.28	27.83	1.00	0.84
P1×P6	24.79	25.00	0.98	0.69
P1×P7	19.97	20.47	0.72	0.45
P1×P8	28.18	28.90	1.01	0.89
P2×P3	19.83	20.47	0.69	0.44
P2×P4	29.04	29.50	1.09	0.95
P2×P5	24.56	25.00	0.91	0.68
P2×P6	23.19	23.36	0.92	0.60
P2×P7	20.74	20.90	0.82	0.48
P2×P8	27.18	27.50	1.05	0.83
P3×P4	25.73	25.85	1.05	0.74
P3×P5	30.06	30.28	1.20	1.02
P3×P6	27.26	27.44	1.09	0.84
P3×P7	25.97	26.51	0.95	0.76
P3×P8	34.15	34.31	1.39	1.31
P4×P5	26.60	27.01	1.00	0.80
P4×P6	26.81	26.94	1.09	0.81
P4×P7	23.04	23.29	0.89	0.60
P4×P8	31.47	31.54	1.32	1.11
P5×P6	27.72	27.80	1.16	0.86
P5×P7	18.80	18.94	0.75	0.40
P5×P8	27.93	28.36	1.05	0.88
P6×P7	27.95	27.97	1.21	0.88
P6×P8	22.07	22.47	0.82	0.55
P7×P8	26.56	26.70	1.08	0.79

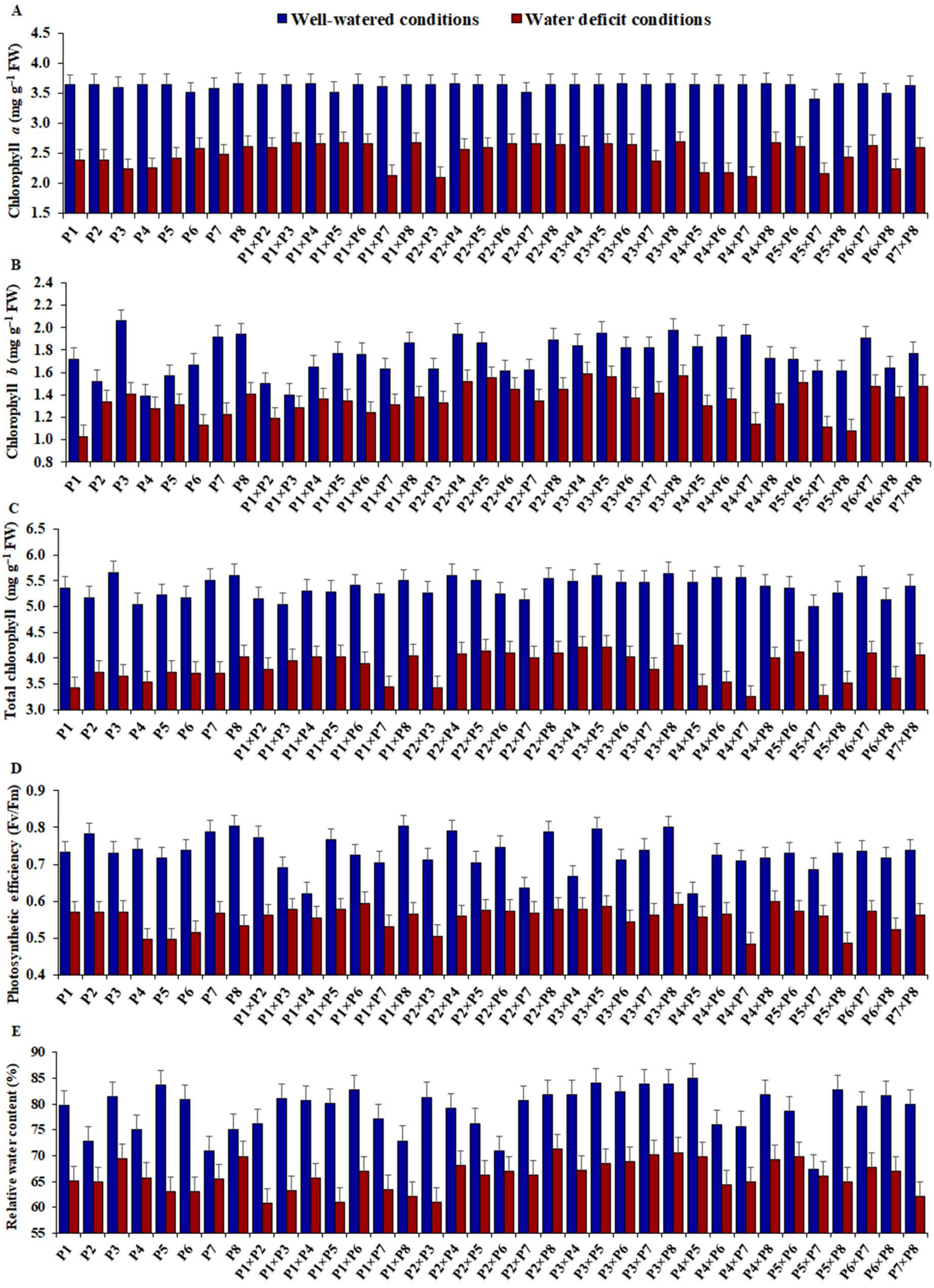

Figure S1. Mean performance of the thirty-six wheat genotypes for chlorophyll *a* (A), chlorophyll *b* (B), total chlorophyll content (C), photosynthetic efficiency (D), and relative water content (E). The bars on the columns represent LSD ($p < 0.05$).

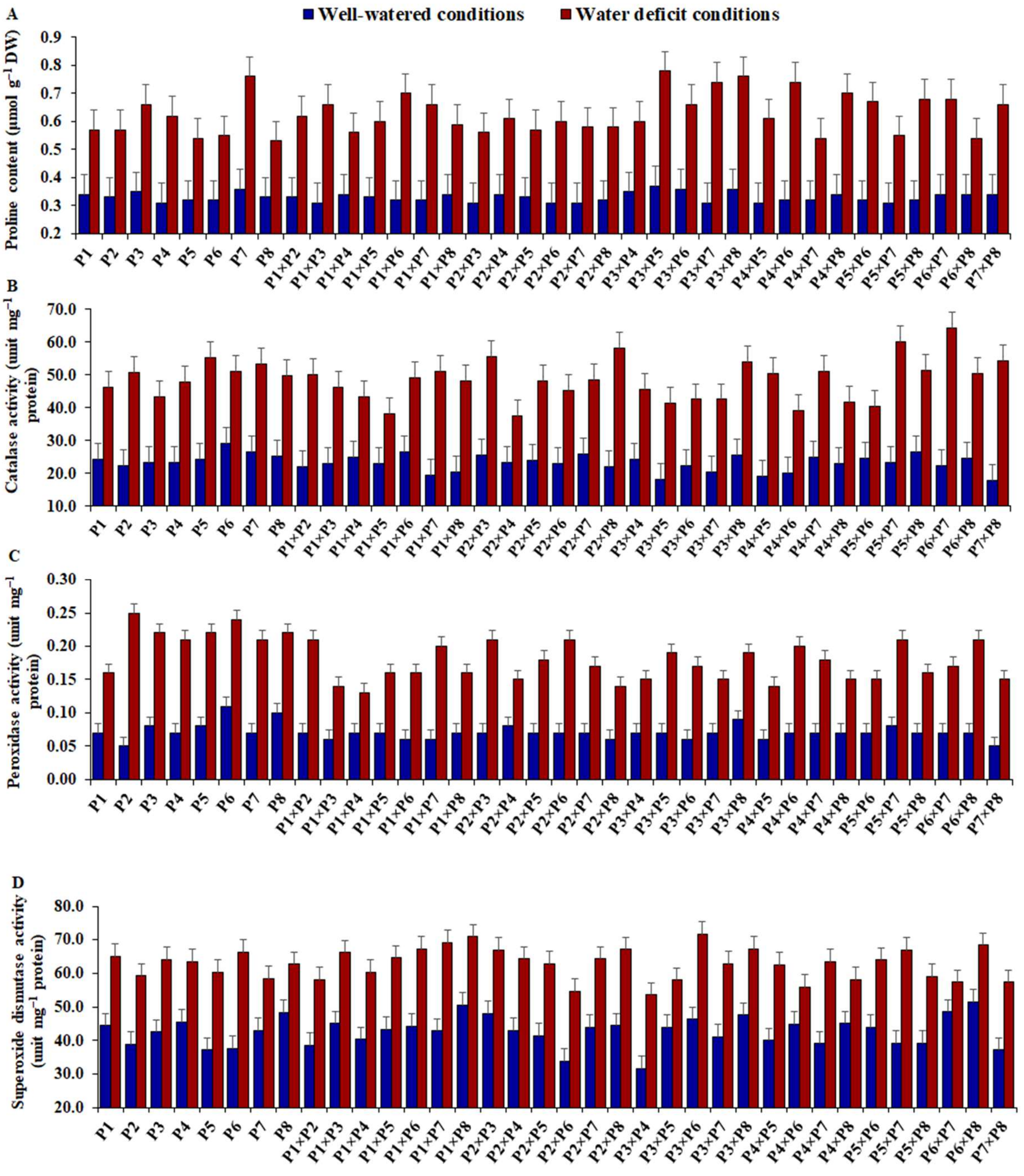

Figure S2. Mean performance of the thirty-six wheat genotypes for proline content (A), catalase activity (B), peroxidase activity (C), and superoxide dismutase activity (D). The bars on the columns represent LSD ($p < 0.05$).

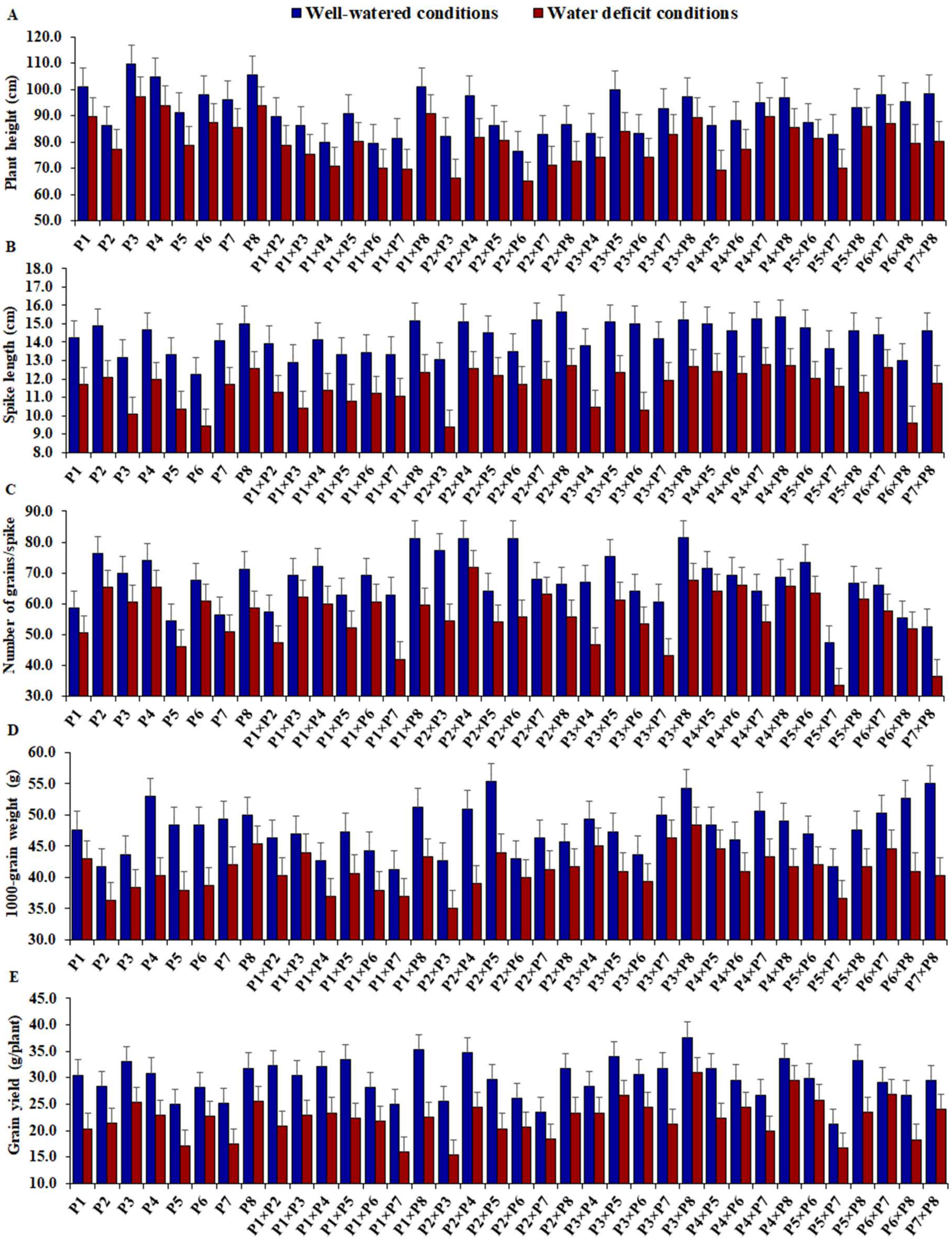

Figure S3. Mean performance of the thirty-six wheat genotypes for plant height (A), spike length (B), number of grains per spike (C), 1000-grain weight (D), and grain yield per plant (E). The bars on the columns represent LSD ($p < 0.05$).

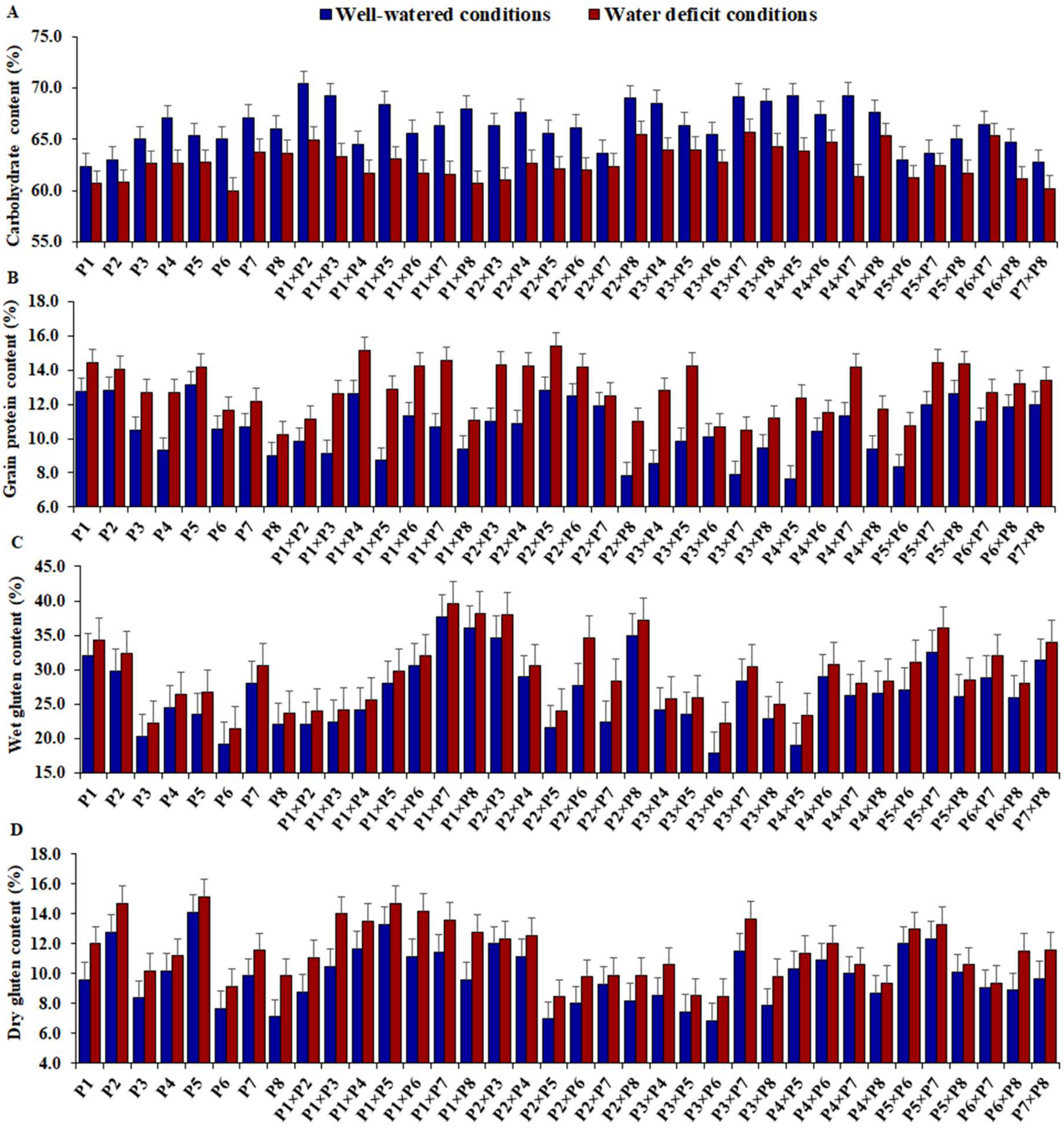

Figure S4. Mean performance of the thirty-six wheat genotypes for carbohydrate content (A), protein content (B), wet gluten content (C), and dry gluten content (D). The bars on the columns represent LSD ($p < 0.05$).

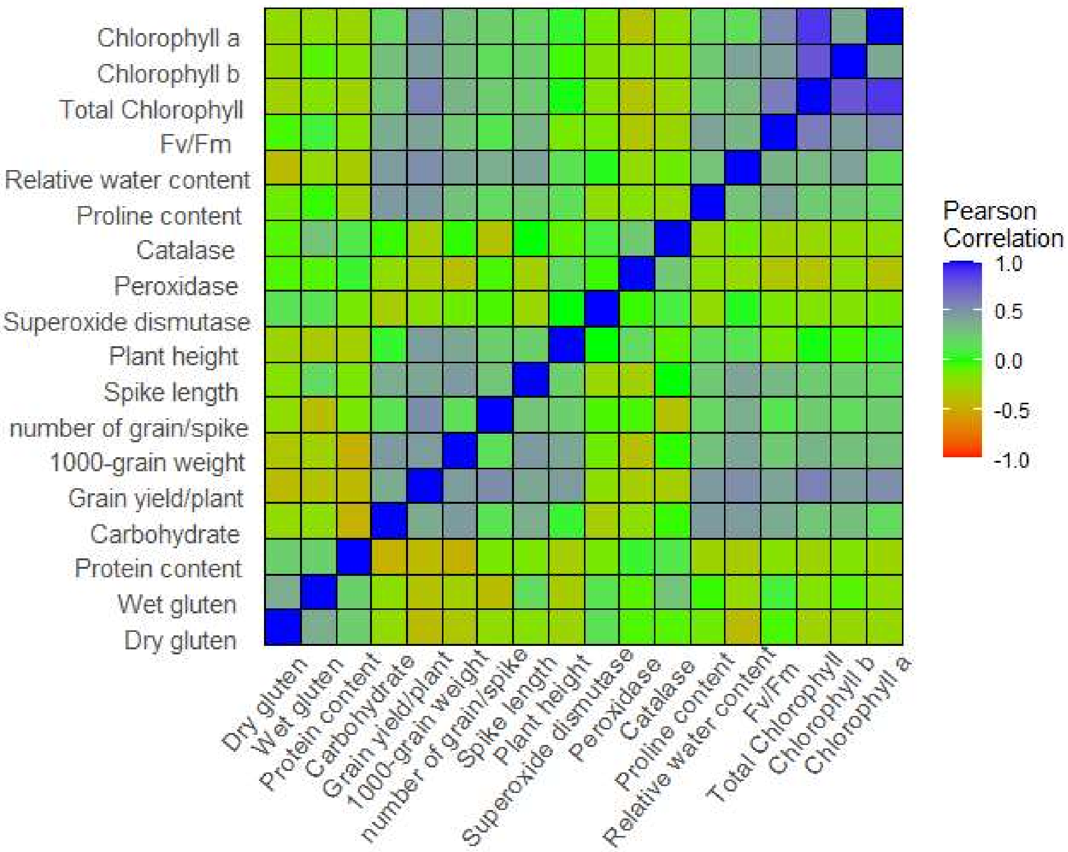

Figure 5. Correlation heatmap of the studied physiological, agronomic, and quality traits.
